# Ion Exchange/Insertion Reactions for Fabrication of Efficient Methylammonium Tin Iodide Perovskite Solar Cells

**DOI:** 10.1002/advs.201903047

**Published:** 2020-03-14

**Authors:** Pengcheng Wang, Fengzhu Li, Ke‐Jian Jiang, Yanyan Zhang, Haochen Fan, Yue Zhang, Yu Miao, Jin‐Hua Huang, Caiyan Gao, Xueqin Zhou, Fuyi Wang, Lian‐Ming Yang, Chuanlang Zhan, YanLin Song

**Affiliations:** ^1^ Inner Mongolia Key Laboratory of Green Catalysis College of Chemistry and Environmental Science Inner Mongolia Normal University Hohhot 010022 China; ^2^ School of Chemical Engineering and Technology Tianjin University Tianjin 300072 P. R. China; ^3^ Key Laboratory of Green Printing Institute of Chemistry Chinese Academy of Sciences Beijing 100190 P. R. China; ^4^ Beijing National Laboratory for Molecular Sciences CAS Key Laboratory of Analytical Chemistry for Living Biosystems Institute of Chemistry Chinese Academy of Sciences Beijing 100190 China

**Keywords:** crystal growth, ion exchange/insertion reaction, lead‐free perovskites, solar cells

## Abstract

The low toxicity, narrow bandgaps, and high charge‐carrier mobilities make tin perovskites the most promising light absorbers for low‐cost perovskite solar cells (PSCs). However, the development of the Sn‐based PSCs is seriously hampered by the critical issues of poor stability and low power conversion efficiency (PCE) due to the facile oxidation of Sn^2+^ to Sn^4+^ and poor film formability of the perovskite films. Herein, a synthetic strategy is developed for the fabrication of methylammonium tin iodide (MASnI_3_) film via ion exchange/insertion reactions between solid‐state SnF_2_ and gaseous methylammonium iodide. In this way, the nucleation and crystallization of MASnI_3_ can be well controlled, and a highly uniform pinhole‐free MASnI_3_ perovskite film is obtained. More importantly, the detrimental oxidation can be effectively suppressed in the resulting MASnI_3_ film due to the presence of a large amount of remaining SnF_2_. This high‐quality perovskite film enables the realization of a PCE of 7.78%, which is among the highest values reported for the MASnI_3_‐based solar cells. Moreover, the MASnI_3_ solar cells exhibit high reproducibility and good stability. This method provides new opportunities for the fabrication of low‐cost and lead‐free tin‐based halide perovskite solar cells.

## Graphical abstract

Tin fluoride and methylammonium iodide are employed as precursors for the fabrication of methylammonium tin iodide (MASnI_3_) film via an ion exchange/insertion reactions approach, and a highly uniform, pinhole‐free perovskite film is obtained with a high concentration of SnF_2_ and a low content of Sn^4+^. The corresponding solar cell exhibits the highest power conversion efficiency of 7.78% with high reproducibility and stability.

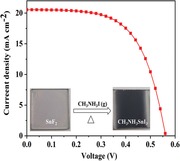

Organic–inorganic hybrid lead halide perovskite solar cells (PSCs) have attracted great attention owing to the excellent optoelectronic properties and easy solution processability of the perovskite materials, and rapid progress on the power conversion efficiencies (PCEs) and stability have been obtained in recent years.^1^, ^2^, ^3^, ^4^ Despite these outstanding achievements, the toxicity of lead (Pb) is considered one of the main obstacles to the commercialization of this technology. Tin (Sn) is among the most promising candidates to replace Pb in the perovskites as both elements belong to the same group of the periodic table with the same outer electron configuration of ns^2^np^2^. In addition, Sn‐based perovskites have the advantages of smaller optical bandgaps and greater charge mobility than the lead‐based perovskites. However, the PCEs obtained so far are far below those of the lead counterparts. The inferior performance is mainly ascribed to the facile oxidation of Sn^2+^ to Sn^4+^ and poor film formability.^5^, ^6^, ^7^, ^8^, ^9^, ^10^ The excess Sn^4+^ ions can produce high carrier concentration and thus more recombination loss, severely deteriorating the semiconductor behavior and device performance. In addition, tin perovskite films usually suffer from low surface coverage due to fast crystallization during the solution deposition.^6^, ^7^, ^8^, ^9^, ^10^

In the past years, great efforts have been devoted to suppress the oxidation for high‐efficiency Sn‐based PSCs.^11^, ^12^, ^13^, ^14^, ^15^, ^16^, ^17^, ^18^, ^19^, ^20^, ^21^, ^22^, ^23^, ^24^, ^25^, ^26^, ^27^, ^28^, ^29^, ^30^, ^31^, ^32^, ^33^, ^34^, ^35^, ^36^ In most of the efficient devices, SnF_2_ or other tin (II) halides is usually incorporated as an indispensible additive during the film formation of the tin perovskites. Especially, SnF_2_ can function as both Sn^2+^ compensator and reducing agent in the Sn‐based perovskite film, and thus has received more great attention. In addition, SnF_2_ can improve film morphology, and adjust energy level positions for efficient charge transfer.^36^ Previously, Liao et al. reported the fabrication of an inverted planar formamidinium tin iodide perovskite (FASnI_3_) device with SnF_2_ additive in varied proportions, and the optimized device showed a high PCE of 6.22%.^34^ In addition, Seok's group reported the homogeneous dispersion of SnF_2_ via the formation of the SnF_2_‐pyrazine complex.^11^ Despite the efforts, the quantity of SnF_2_ additive is still limited due to severe phase segregation in the perovskite film deposited via the conventional solution process. On the other hand, excess SnI_2_ was incoporated in Sn‐based perovskite films during the solution deposition, resulting in an improved PCE of 4.81%.^26^ In addition, excess SnI_2_ could be incorporated in the perovskite film using a two‐step process, where spin‐coated SnI_2_ reacted with methylammonium iodide (MAI) vapor to yield a MASnI_3_ film with a certain amount of unreacted SnI_2_, and the resulting film exhibited improved air stability due to the presence of the excess Sn^2+^.^27^ In addition, the crystallization of Sn‐based perovskites is much faster than that of the Pb analogs during the solution deposition because of the lower solubility of SnI_2_ than PbI_2_, resulting in abundant trap states in the Sn‐based perovskite flms. Therefore, it is necessary to develop new and more effective strategies to reduce the background carrier density and improve the performance of the tin perovskite solar cells.

Different from the conventional solution or solid–gas reaction approaches, where high‐purity and unstable SnI_2_ was employed as tin source, in this work, SnF_2_ and MAI were employed as precursors for the fabrication of MASnI_3_ perovskite films via ion exchange/insertion reactions approach. In this way, a highly uniform, pinhole‐free MASnI_3_ perovskite film was achieved with a large amount of SnF_2_ and a low content of Sn^4+^. The corresponding PSCs afforded a champion power conversion efficiency (PCE) of 7.78% with high reproducibility and good stability.

**Figure** **1**a illustrates the basic steps for the fabrication of the MASnI_3_ film. Detailed procedures are provided in Experimental Section. Briefly, 1.5 m SnF_2_ was dissolved in poly(3,4‐ethylenedioxythiophene) polystyrene sulfonate (PEDOT:PSS) aqueous solutions, and then spin‐coated onto an indium tin oxide (ITO) glass covered with a thin PEDOT:PSS layer. After drying, the SnF_2_/PEDOT:PSS film was moved in N_2_‐filled box, and loaded face down on a Petri dish on a hotplate, where MAI powder was put well at the bottom of the film. At 140 °C, the color of the pristine film was changed gradually from white to light brown after 2 min, then dark brown in 10 min, and finally to black in 20 min. Clearly, MASnI_3_ is formed in the reactions between SnF_2_ and evaporated MAI. The conversion may be proceeded via ion exchange/insertion reactions as follows:

(1) $${\text{SnF}}_{2}+2{\text{CH}}_{3}{\text{NH}}_{3}\text{I}\to {\text{SnI}}_{2}+2{\text{CH}}_{3}{\text{NH}}_{3}\text{F}$$

(2) $${\text{CH}}_{3}{\text{NH}}_{3}\text{F}\to {\text{CH}}_{3}{\text{NH}}_{2}\;\;↑\;\;+\text{HF}↑$$

(3) $${\text{SnI}}_{2}+{\text{CH}}_{3}{\text{NH}}_{3}\text{I}\to {\text{CH}}_{3}{\text{NH}}_{3}{\text{SnI}}_{3}$$

**Figure 1:**
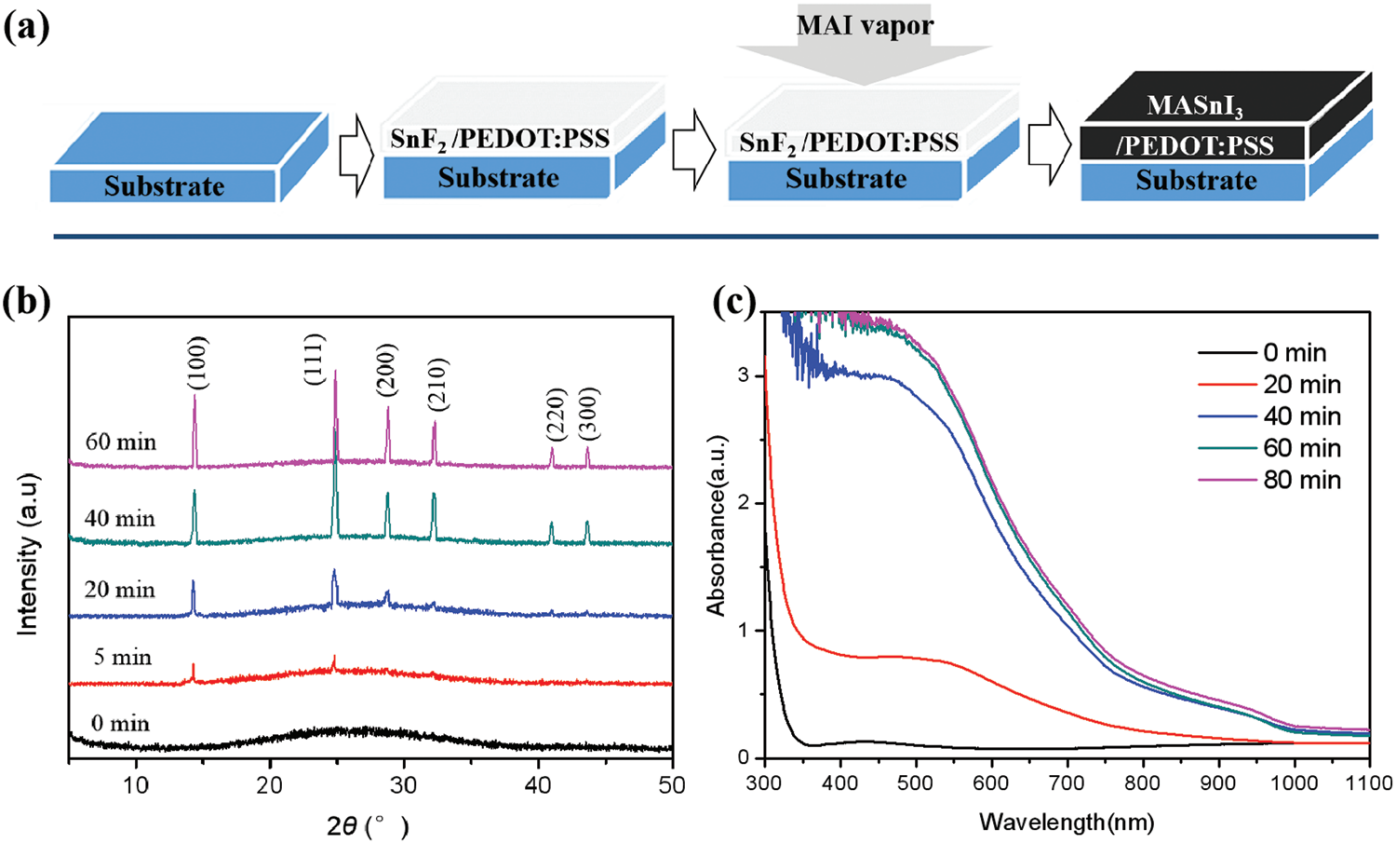
a) Schematic diagram of MASnI_3_ perovskite film via ion exchange/insertion reaction process, b) XRD and c) UV–vis spectra of the conversion in the reaction at the different time intervals.

In the first step, ion exchange reaction is involved between solid‐state SnF_2_ and gaseous CH_3_NH_3_I, generating the intermediate SnI_2_ and byproduct CH_3_NH_3_F. The CH_3_NH_3_F is assumed to be lost via evaporation in the second step. In the third step, gaseous MAI is further inserted into SnI_2_ to form MASnI_3_, similar to the vapor‐assisted process reported previously.^37^ The ion exchange/insertion reaction process is very similar to that for MAPbI_3_ using PbO and MAI as precursors in our previous report.^38^

The X‐ray diffraction spectra were recorded for the SnF_2_/PEDOT:PSS films in the reaction at the different time intervals. As shown in Figure 1b, the pristine film is amorphous, as evident by a broad bump at 2θ = 20°–35°, which is in contrast with the sharp diffraction peaks from the SnF_2_ film directly deposited from the aqueous solution (Figure S1, Supporting Information). The result implies that PEDOT:PSS can effectively prevent the crystallization of SnF_2_ for the amorphous film, favoring the following ion exchange/insertion reactions between SnF_2_ and gaseous MAI. The converted SnI_2_ can serve as nucleation centers, and subsequently react with gaseous MAI to form MASnI_3_ film, similar to the processes for the solution‐deposited MAPbI_3_ film using PbCl_2_ and MAI (PbCl_2_ + 3MAI) as precursors reported previously,^39^ enabling a more controllable film formation process. After 5 min, two weak peaks were observed at 14.3° and 24.9°, corresponding to the (100) and (110) planes of MASnI_3_ pseudocubic phase, respectively. With the time increased from 5 to 40 min, the new peaks for (200), (210), (220), and (300) planes from the MASnI_3_ phase appeared, and became stronger with the time, indicative of a strong recrystallization process and a more favorable packing arrangement. Figure 1c shows the corresponding optical absorption spectra of the film, which are consistent with the X‐ray diffraction (XRD) spectra. Steady‐state PL spectra were recorded for the MASnI_3_ films fabricated on glass substrates with an aqueous SnF_2_ solution and a SnF_2_/PEDOT:PSS solution, respectively, followed with the cation displacement reaction, as shown in Figure S2, Supporting Information. We observed that the PL intensity of the MASnI_3_ film with PEDOT:PSS is much lower than that of the film without PEDOT:PSS. The significantly reduced PL intensity can be attributed to the quenching effect (improved charge collection) caused by the hole transfer promoted by the PEDOT:PSS underneath the converted MASnI_3_ layer.

To get insight into the ion exchange/insertion reaction process, the microstructure and surface morphology were examined by scanning electron microscopy (SEM) as shown in **Figure** **2**. The pristine film is uniform with complete surface coverage, in consistent with the amorphous structure. The high‐magnifcation image shows that the film is covered with fine mesh cracks, which may favor the ion exchange reaction between SnF_2_ and gaseous MAI. When the film is exposed to MAI vapor for 5 min, a large number of tiny and angular MASnI_3_ crystals were observed, embedded homogeneously in the amorphous film. These small crystal “seeds” grow up with the extending reaction time, followed with the depletion of SnF_2_. After 40 min, the crystals were grown up to ≈300 mm in size. Interestingly, the crystal size was further increased to ≈600 nm after an additional 20 min, and the resultant MASnI_3_ film is continuous and compact without pinholes. The crystals seemed to have no change in size with prolongation of reaction time. It should be noted that the crystal size is larger than the thickness of the film so that there is minimized grain boundaries in the charge collection direction of the perovskite films, which may contribute to a high efficiency in the perovskite solar cells. Additionally, the film thickness was increased to ≈300 nm from ≈200 nm for the pristine SnF_2_/PEDOT:PSS film (Figure S3, Supporting Information), probably due to volume expansion from intercalation of MAI.

**Figure 2:**
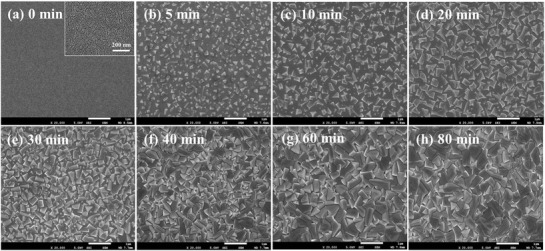
a) Surface SEM images of the pristine SnF_2_/PEDOT:PSS film, and treated by the ion exchange/insertion reaction for b) 5 min, c) 10 min, d) 20 min, e) 30 min, f) 40 min, g) 60 min, and h) 80 min.

To characterize the elemental composition and thus its functional components, energy‐dispersive X‐ray spectroscopy (EDAX) was performed for the films converted at the different time intervals, and the relevant atomic ratios of the main elements (Sn, F, and I) are listed in Table S1, Supporting Information. It was found that the iodine content in the films was gradually increased with prolonging the reaction time, and meanwhile the fluorine content decreased accordingly, indicative of the ongoing process of the ion exchange reaction. After 60 min, the ratio of Sn, I, and F is about 1:1.7:0.8 in the film, implying that the converted MASnI_3_ coexists with SnF_2_ at the mole ratio of ≈3:2 in the film, assuming that the I and F exist in the forms of MASnI_3_ and SnF_2_, respectively. In the MASnI_3_ film, the SnF_2_ content is about seven times higher than that of the conventional one‐step deposited MASnI_3_ film doped with 10 mol% SnF_2_. By the approach, a continuous and compact MASnI_3_ film can be obtained with a large amount of SnF_2_ additive, which could not be realized by the conventional solution deposition method. For further insight of the distribution of SnF_2_ in the film, time‐of‐flight‐secondary ion mass spectroscopy (ToF‐SIMS) measurement was performed. The ToF‐SIMS elemental depth profiles of iodine and fluorine ions were recorded for the converted perovskite film (60 min), as shown in **Figure** **3**a. Both the ions are dispersed through the film with high concentrations beyond the upper detecting limit, consistent with the EDAX result. In addition, it was found that the film surface is iodine‐rich, and the bottom is fluorine‐rich. In the case of the control sample doped with 10 mol% SnF_2_, the distribution of fluorine is inhomogeneous in the depth profile with high concentrations only in the surface and bottom regions, as shown in Figure 3b. In the bulk region, the intensity of fluorine ion for the control sample is about two orders of magnitude lower than that for the current MASnI_3_ film.

**Figure 3:**
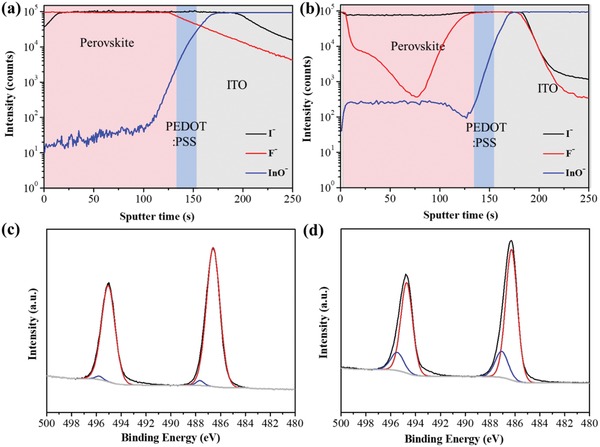
a,c) ToF‐SIMS elemental depth profiles for iodine and fluorine, and high‐resolution XPS spectra (Sn 3d) for the MASnI_3_ films based on ion exchange/insertion approach and b,d) the conventional one‐step solution method with 10 mol% of SnF_2_. In (c) and (d), black line, measured results; red line, Sn^2+^ state; blue line, Sn^4+^ state; gray line, background.

To clarify the role of SnF_2_ in suppressing the oxidation of Sn^2+^ to Sn^4+^, the X‐ray photoelectron spectra (XPS) were recorded to analysis the Sn states on the MASnI_3_ films based on the ion exchange/insertion reactions and the conventional one‐step deposition approach. Both the films were etched with Ar^+^ sputtering for 30 s for the detection of the bulky MASnI_3_ films. The XPS spectra for the two samples can be deconvoluted into two components associated with Sn^2+^ and Sn^4+^ species, as shown in Figure 3c,d. The MASnI_3_ film reported here shows two sharp peaks located at 495.0 and at 486.6 eV for Sn 3d_3/2_ and Sn 3d_5/2_, respectively, and the amounts of Sn^2+^ and Sn^4+^ were about 98% and 2%. In contrast, the control sample presents two broad peaks, and the amounts of Sn^2+^ and Sn^4+^ are calculated to be 82% and 18%, respectively. The results indicate that the presence of SnF_2_ with high concentration can effectively suppress the oxidation of Sn^2+^ to Sn^4+^, and thus decrease Sn vacancy concentration in the MASnI_3_ film, which can improve the performance and stability of the photovoltaic devices. In addition, the XPS spectra of the pristine SnF_2_/PEDOT:PSS film and the corresponding converted MASnI_3_ film were measured as shown in Figure S4, Supporting Information. In the former film, the O 1s peak can be detected with the element contents of ≈30 at%. The high content of oxygen could come from the PEDOT:PSS and impurity SnO/Sn(OH)_2_ forming during the film annealing. After the conversion, the oxygen content dramatically decreased to ≈9 at%, indicating that PEDOT:PSS could remain underneath the converted MASnI_3_ layer after the treatment.

The tin perovskite films were subsequently used for photovoltaic device fabrication. The tin perovskite films with various reaction times are labeled as MAI‐20, MAI‐40, MAI‐60, and MAI‐80, where the numbers 20, 40, 60, and 80 represent different times (minute) for the ion exchange/insertion reactions. For comparison, the control device was prepared with the MASnI_3_ perovskite film deposited by the conventional solution method with precursors MAI and SnI_2_ doped with 10 mol% SnF_2_. All the devices have a planar heterojunction structure consisting of ITO/PEDOT:PSS/MASnI_3_/PC_61_BM/BCP/Ag, where PEDOT:PSS and PC_61_BM ([6,6]‐phenyl C_61_ butyric acid methyl ester) serve as the hole transport and electron transport layers, respectively, and a thin layer of 2,9‐dimethyl‐4,7‐diphenyl‐1,10‐phenanthroline (BCP) enhances the contact between the PC_61_BM and the Ag electrode. The detailed device fabrication process is described in Supporting Information.

**Table** **1** listed the photovoltaic parameters of the best solar cells, including open‐circuit voltage (*V*_oc_), short‐circuit current density (*J*_sc_), and fill factor (FF). The performance of MAI‐20 is relatively inferior, with a best PCE of 1.39%, probably due to low content of MASnI_3_ and poor film coverage in the active layer. With prolonging the reaction time for the MASnI_3_, the devices show improved *J*_SC_ values and FF, leading to improved overall PCEs. The highest PCE for champion MAI‐60 device yields a PCE of 7.78% with a *J*_sc_ of 20.68 mA cm^‐2^, a *V*_oc_ of 0.57 V, and a FF of 0.66. The PCE is among the highest values reported for the MASnI_3_‐based solar cells, and is more than four times higher than that for the control device with the MASnI_3_ layer doped with 10 mol% of SnF_2_. It was found that the *V*_oc_ values slightly decreased for the devices with the MASnI_3_ films prepared with the reaction time extending. As mentioned above, the SnF_2_ content is decreased with the time in the ion exchange/insertion reactions. The result implies that the presence of high content SnF_2_ can effectively suppress the oxidation of Sn^2+^ to Sn^4+^, and decrease carrier concentration and thus more recombination loss, leading to high *V*_oc_ values and improved photovoltaic performance. With the reaction time extending, the performance of MAI‐80 is decreased due to the decrease of all the parameters, probably due to the decrease of SnF_2_ content in the active layer. **Figure** **4**a shows the photocurrent density versus voltage (*J–V*) curves of the champion device MAI‐60 and the best control device, and Figure 4b presents the corresponding incident photon‐to‐current conversion efficiency (IPCE). The IPCE spectrum displays a high average value between 400 and 1000 nm wavelength range, consistent with the absorption profile, and the value of integrated *J*_sc_ (20.25 mA cm^−2^) agrees well with that from the *J–V* measurement. The PCE histogram in Figure 4c presents an average PCE of 7.05% from 30 devices, showing high reproducibility. The MASnI_3_‐based devices show low hysteresis in forward and reverse directions with various scan rates, as shown in Figures S5 and S6, Supporting Information. In addition, the device stability test was carried out for unencapsulated PSCs in continuous operation under 1‐sun illumination at 25 °C in N_2_ atmosphere, as shown in Figure 4d. The PCE of MAI‐60 is slowly decreased with time, and maintained ≈70% of its original efficiency over 200 h, while the control device shows poor stability with negligible PCE after 200 h. Consequently, these results clearly demonstrate that the high content SnF_2_ can effectively improve the performance and stability of the device.

**Table 1:** Best solar cell performance parameters, of solar cells based on MASnI_3_ fabricated via ion exchange/insertion approach with different reaction times

Reaction time [min]	*J*_sc_ [mA cm^2^]	*V*_oc_ [V]	FF	PCE [%]
20	6.52	0.61	0.35	1.39
40	17.47	0.60	0.52	5.45
60	20.68	0.57	0.66	7.78
80	18.33	0.53	0.65	6.31
Control device	12.36	0.32	0.41	1.62

**Figure 4:**
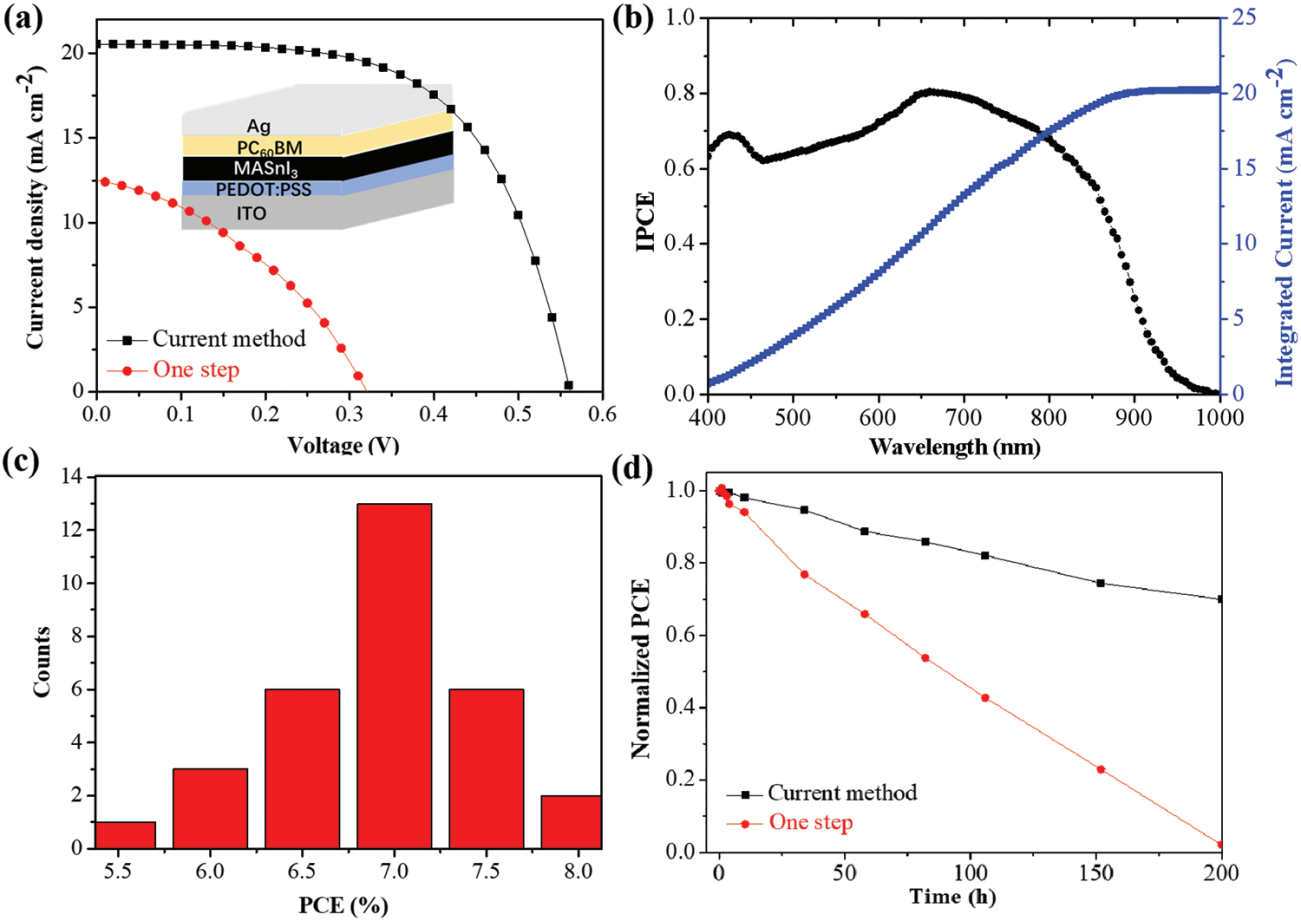
a) *J*–*V* curves of the best devices with MASnI_3_ films prepared by current ion exchange/insertion method and the one‐step solution method, respectively. b) IPCE spectrum for the device via ion exchange/insertion method. c) Histograms of PCEs for 30 devices via ion exchange/insertion method. d) PCE evolution of unencapsulated PSCs in continuous operation under 1‐sun illumination at 25 °C in N_2_ atmosphere.

In summary, for the first time, an ion exchange/insertion approach is developed for the fabrication of MASnI_3_ perovskite film using tin fluoride and MAI as precursors. The resulting film is uniform, continuous, and pinhole free with a controllable amount of SnF_2_, which is well dispersed in the depth profile of the film. The presence of high content SnF_2_ can effectively suppress the oxidation of Sn^2+^ to Sn^4+^. With the MASnI_3_ film as light absorber in the planar heterojunction solar cell, a high power conversion efficiency of 7.78% was obtained, which is among the best values for MASnI_3_‐based perovskite solar cells. Further improvement of the efficiency could be expected through optimization of the fabrication process parameters. Moreover, the MASnI_3_‐based perovskite solar cells shows good environmental stability, and no toxic organic solvents is used to dissolve the precursors in the fabrication. Therefore, this novel interdiffusion process could provide an environmental benign protocol for the fabrication of tin‐based PCSs with high efficiency and stability.

## Experimental Section

##### Perovskite Film Fabrication

The ITO‐coated glass substrates were cleaned successively with detergent, deionized water, ethanol, and acetone in an ultra‐sonication bath for 25 min, respectively, and then treated with UV–ozone for 30 min. Then, aqueous PEDOT:PSS were filtered with a 0.22 µm PTFE filter, and spin‐coated onto the ITO substrate at 4000 rpm for 30 s, followed with annealing at 150 °C for 15 min. SnF_2_ (1.5 m) was dissolved in aqueous PEDOT:PSS and stirred for 5 h. The PEDOT:PSS/SnF_2_ solutions were spin‐coated on the top of the preceding PEDOT:PSS layer at 2000 rpm for 30 s. After annealing at 100 °C for 20 min, the substrates were transferred into a nitrogen‐filled glove box, and placed face down on a petri dish on a hotplate, where the sample was set on two glass (2 mm thickness) with a spacing of 13 mm, and 200 mg MAI powder was uniformly dispersed under the sample. All these procedures were performed in a N_2_‐filled glove box, and the hotplate was kept at 140 °C for the reactions. For comparison, the one‐step MASnI_3_ layer films were spin‐coated from a precursor solution comprising MAI (1 m), SnI_2_ (1 m), and SnF_2_ (0.1 m) in mixed solvents of DMF (800 µL) and DMSO (200 µL) at 5000 rpm for 60 s. 150 µL anti‐solvent (chlorobenzene) was dripped onto the substrate after 20 s of the coating process. The perovskite films were then annealed at 100 °C for 10 min on a hotplate. Before the film deposition, the solutions were stirred for 1 h and filtered with a 0.22 µm PTFE filter before use.

##### Device Fabrication

After the fabrication of perovskite layer, 20 mg PCBM dissolved in 1 mL chlorobenzene was deposited on the perovskite film by spin‐coating at 2000 rpm for 30 s, then annealed at 70 °C for 5 min, followed with the coating of BCP from its saturated solution in isopropanol at 4000 rpm for 30 s and then annealing at 70 °C for 5 min. Finally, an 80 nm Ag electrode was deposited via thermal evaporation at a constant rate of 0.02 nm s^−1^.

##### Film Characterization

The XRD patterns of the prepared films were recorded using an X‐ray diffractometer (Rigaku, D/MAX RINT‐2500) with Cu K radiation (=1.54 Å) at a speed of 5° min^−1^. The absorption spectra were collected using a UV–vis spectrometer (Shimadzu, UV‐1800 UV–vis Spectrophotometer) in the wavelength range of 300–1000 nm. The surface and cross‐sectional morphologies of the films as well as the thicknesses were analyzed by using a JEM‐7500F field‐emission scanning electron microscope (SEM). EDAX of the thin films were investigated by JEM‐7500F. X‐ray photoelectron spectroscopy (XPS) were performed on the Thermo Scientific ESCALAB 250Xi using 200 W monochromated Al Kα radiation, and 500 µm X‐ray spot was used for XPS analysis. The ToF‐SIMS measurements were performed in a ToF‐SIMS V instrument (IONTOF GmbH, Münster, Germany). During analysis, dual‐beam depth profiling in an interlaced mode was used. A pulsed 30 keV Bi^+^ ion beam (10 kHz, 1.08 pA current) was used for scanning on an area of 100 × 100 µm^2^ on the sample surface. A 1 keV Cs^+^ with a 68 nA beam current was used for the sputtering with a crater size of 350 × 350 µm^2^. A flood gun with a current of ≈2 µA was used during analysis for charge compensation.

##### Solar Cell Characterizations

Current–voltage (*J–V*) characteristics were measured in the N_2_‐filled glove box. The curves were recorded by applying an external potential bias to the cell while recording the generated photocurrent with a Keithley model 2400 digital source meter. The light source was a 300 W collimated xenon lamp (Newport) calibrated with the light intensity to 100 mW cm^−2^ under AM 1.5 G solar light conditions by a certified silicon solar cell. The *J–V* curve was recorded by the reverse scans with a rate of 100 mV s^−1^. The active area of 9 mm^2^ was determined by metal shadow mask. The IPCE was performed outside for the enveloped solar cells using a commercial setup (PV‐25 DYE, JASCO). A 300 W xenon lamp was employed as a light source for the generation of a monochromatic beam. IPCE spectra were recorded using monochromatic light without white light bias. Calibrations were performed with a standard silicon photodiode.

## Conflict of Interest

The authors declare no conflict of interest.

## Supporting information

Supporting Information
