# Supplementary material for: Ion Exchange/Insertion Reactions for Fabrication of Efficient Methylammonium Tin Iodide Perovskite Solar Cells
Source: Adv Sci (Weinh). 2020 Mar 14;7(9):1903047. doi: 10.1002/advs.201903047 (PMC7201265; doi:10.1002/advs.201903047)
Supplement: Supplementary file 1 — Supporting Information [file ADVS-7-1903047-s001.pdf]

Copyright WILEY-VCH Verlag GmbH & Co. KGaA, 69469 Weinheim, Germany, 2020.

ADVANCED SCIENCE
Open Access

Supporting Information

for *Adv. Sci.*, DOI: 10.1002/advs.201903047

Ion Exchange/Insertion Reactions for Fabrication of Efficient Methylammonium Tin Iodide Perovskite Solar Cells

Pengcheng Wang, Fengzhu Li, Ke-Jian Jiang, Yanyan Zhang, Haochen Fan, Yue Zhang, Yu Miao, Jin-Hua Huang, Caiyan Gao, Xueqin Zhou, Fuyi Wang, Lian-Ming Yang, Chuanlang Zhan,* and YanLin Song**

Supporting Information

Ion Exchange/Insertion Reactions for Fabrication of Efficient Methylammonium Tin Iodide Perovskite Solar Cells

Pengcheng Wang,[†] Fengzhu Li,[†] Ke-Jian Jiang, Yanyan Zhang, Haochen Fan, Yue Zhang, Yu Miao, Jin-Hua Huang, Caiyan Gao, Xueqin Zhou, Fuyi Wang, Lian-Ming Yang, Chuanlang Zhan*, YanLin Song**

Experimental Section

Perovskite Film Fabrication: The ITO-coated glass substrates were cleaned successively with detergent, deionized water, ethanol and acetone in an ultra-sonication bath for 25 min, respectively, and then treated with UV-ozone for 30 min. Then, aqueous PEDOT:PSS were filtered with a 0.22 μm PTFE filter, and spin-coated onto the ITO substrate at 4000 rpm for 30 s, followed with annealing at 150 °C for 15 min. SnF_2 (1.5 M) dissolved in aqueous PEDOT:PSS and stirred for 5 h. The PEDOT:PSS/SnF_2 solutions were spin-coated on the top of the preceding PEDOT:PSS layer at 2000 rpm for 30 s. After annealed at 100 °C for 20 min, the substrates were transferred into a nitrogen-filled glove-box, and placed face down on a petri dish on a hotplate, where the sample was set on two glass (2 mm thickness) with a spacing of 13mm, and 200 mg MAI powder was uniformly dispersed under the sample. All these procedures were performed in a N_2-filled glovebox, and the hotplate was kept at 140 ℃ for the reactions. For comparison, the one-step $MASnI_3$ layer films were spin-coated from a precursor solution comprising MAI (1M), SnI_2 (1M) and SnF_2 (0.1M) in mixed solvents of DMF (800 μL) and DMSO (200 μL) at 5000 rpm for 60 s. 150 μL anti-solvent (chlorobenzene) was dripped onto the substrate after 20 seconds of the coating process. The perovskite films were then annealed at 100 °C for 10 min on a hotplate. Before the film deposition, the solutions were stirred for 1 h and filtered with a 0.22 μm PTFE filter before use.

Device Fabrication: After the fabrication of perovskite layer, 20 mg PCBM dissolved in 1 mL chlorobenzene was deposited on the perovskite film by spin-coating at 2000

rpm for 30 s, then annealed at 70 °C for 5 min, followed with the coating of BCP from its saturated solution in isopropanol at 4000 rpm for 30 s and then annealing at 70 °C for 5 min. Finally, an 80 nm Ag electrode was deposited via thermal evaporation at a constant rate of 0.02 nm/s.

Film Characterization: The X-ray diffraction (XRD) patterns of the prepared films were recorded using an X-ray diffractometer (Rigaku, D/MAX RINT-2500) with Cu K radiation (= 1.54 Å) at a speed of 5° min^{-1}. The absorption spectra were collected using a UV-vis spectrometer (SHIMADZU, UV-1800 UV-vis Spectrophotometer) in the wavelength range of 300~1000 nm. The surface and cross-sectional morphologies of the films as well as the thicknesses were analyzed by using a JEM-7500F field-emission scanning electron microscope (SEM). Energy-dispersive X-ray spectroscopy (EDAX) of the thin films were investigated by JEM-7500F. X-ray photoelectron spectroscopy (XPS) were performed on the Thermo Scientific ESCA Lab 250Xi using 200 W monochromated Al Kα radiation, and 500 μm X-ray spot was used for XPS analysis. The ToF-SIMS measurements were performed in a ToF-SIMS V instrument (ION-TOF GmbH, Münster, Germany). During analysis, dual-beam depth profiling in an interlaced mode was used. A pulsed 30 keV Bi+ ion beam (10 kHz, 1.08 pA current) was used for scanning on an area of 100×100 μm^2 on the sample surface. A 1 keV Cs+ with a 68 nA beam current was used for the sputtering with a crater size of 350×350 μm^2. A flood gun with a current of ~2 μA was used during analysis for charge compensation.

Solar cell characterizations: Current–voltage (*J*–*V*) characteristics were measured in the N_2-filled glove box. The curves were recorded by applying an external potential bias to the cell while recording the generated photocurrent with a Keithley model 2400 digital source meter. The light source was a 300 W collimated xenon lamp (Newport) calibrated with the light intensity to 100 mW/cm^2 under AM 1.5 G solar light conditions by a certified silicon solar cell. The *J*–*V* curve was recorded by the reverse scans with a rate of 100 mV s^{-1}. The active area of 9 mm^2 was determined by metal shadow mask. The incident photon-to-current conversion efficiency (IPCE) was performed outside for the enveloped solar cells using a commercial setup (PV-25 DYE, JASCO). A 300 W Xenon lamp was employed as a light source for the generation of a monochromatic beam. IPCE spectra were recorded using monochromatic light without white light bias. Calibrations were performed with a standard silicon photodiode.

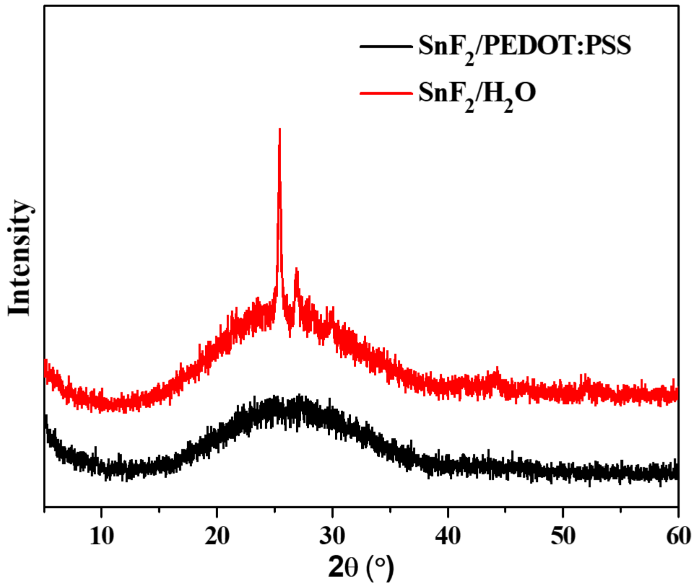

Figure S1. X-ray diffraction patterns (XRD) of SnF_2 films deposited from H_2O solutions with and without PEDOT:PSS.

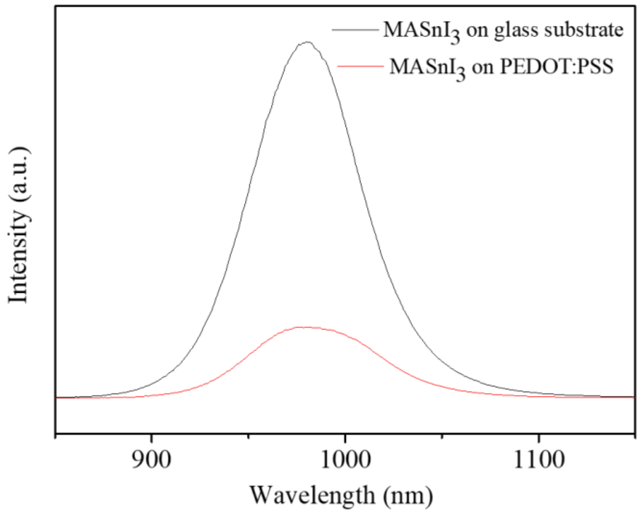

Figure S2. Steady-state PL spectra were recorded for the $MASnI_3$ films fabricated on glass substrates with an aqueous SnF_2 solution (black line) and a SnF_2/PEDOT:PSS solution (red line), respectively, followed by the cation displacement reaction.

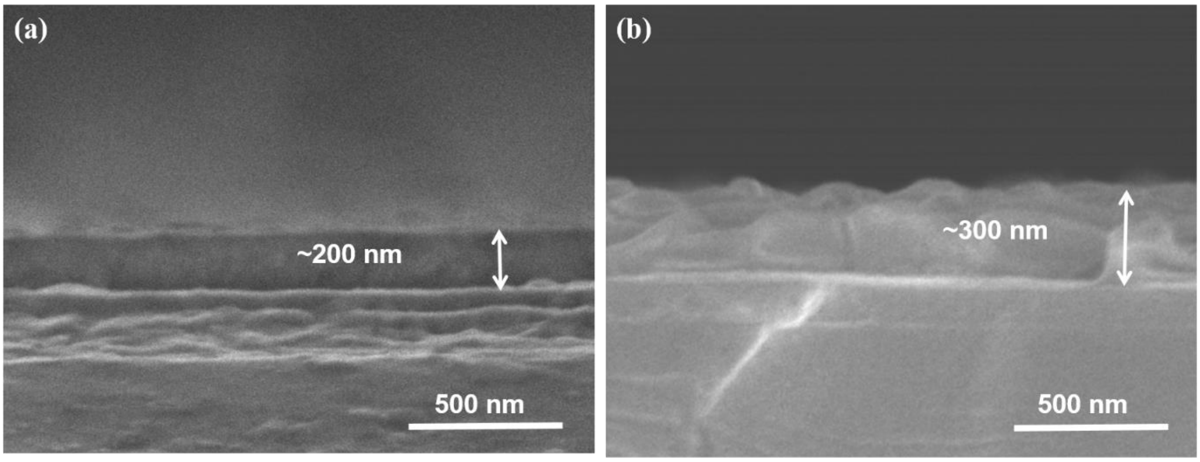

Figure S3. Cross-sectional SEM images of the pristine SnF_2/PEDOT:PSS film and the corresponding converted $MASnI_3$ film.

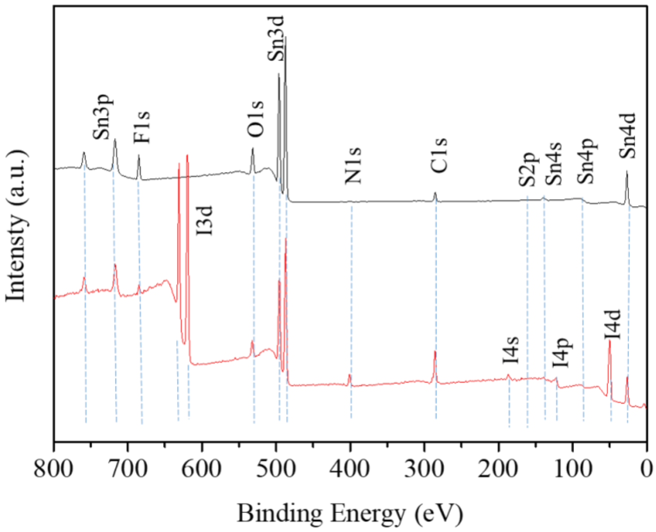

Figure S4. XPS spectra of the SnF_2/PEDOT:PSS film (upper, black line) and the corresponding converted $MASnI_3$ film (below, red line). In the former film, the O1s peak can be detected with the element contents of ~ 30 atomic %. The high content of oxygen could come from the PEDOT:PSS and impurity SnO/$Sn(OH)_2$ forming during the film annealing. After the conversion, the oxygen content dramatically decreased to ~ 9 atomic %, indicating that PEDOT:PSS could remain underneath the converted $MASnI_3$ layer.

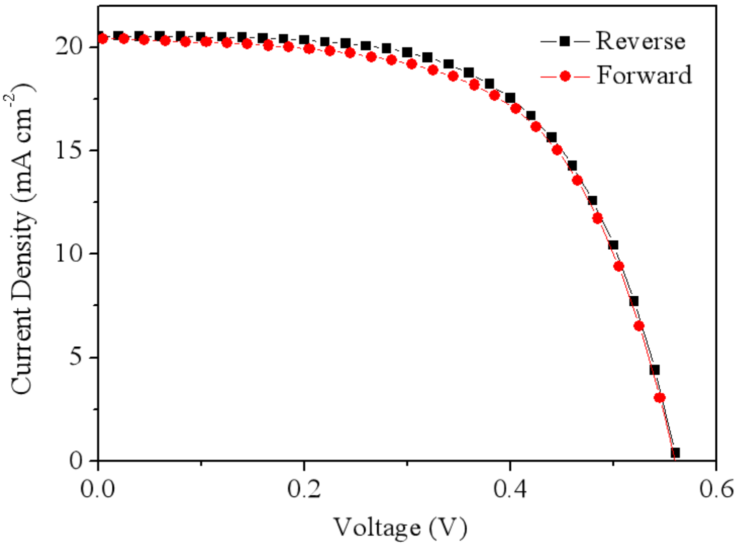

Figure S5. *J-V* characteristic of the $MASnI_3$-based device recorded by the reverse and forward scans with a rate of 100 mV s^{-1}.

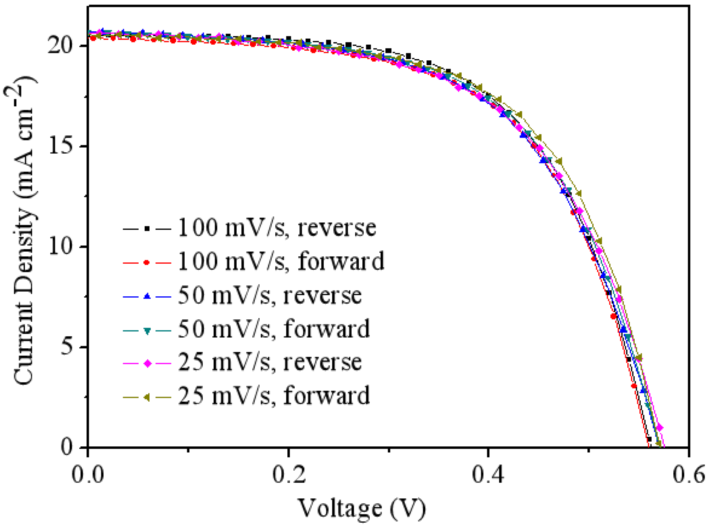

Figure S6 *J-V* characteristics of the $MASnI_3$-based device scanned in forward and reverse directions with various scan rates, which shows no significant hysteresis.

Table S1. EDAX determined atomic ratios of Sn, I and F for films at different time internals in the ion exchange/insertion reactions.

Reaction time / min	Sn	I	F
0	1	0	2.1
5	1	0.3	1.8
10	1	0.9	1.4
20	1	1.2	1.2
30	1	1.3	1.1
40	1	1.6	0.9
60	1	1.7	0.8
80	1	2.1	0.5
